# Allele frequency of pathogenic variants causing acid sphingomyelinase deficiency and Gaucher disease in the general Japanese population

**DOI:** 10.1038/s41439-024-00282-z

**Published:** 2024-06-12

**Authors:** Shuhei Sako, Kimihiko Oishi, Hiroyuki Ida, Eri Imagawa

**Affiliations:** Department of Pediatrics, The Jikei University School of Medicine, Tokyo, Japan

**Keywords:** Metabolic disorders, Paediatrics

## Abstract

Acid sphingomyelinase deficiency (ASMD) and Gaucher disease (GD) are lysosomal storage disorders associated with hepatosplenomegaly and thrombocytopenia. The incidences of ASMD and GD are known to be particularly high in the Ashkenazi Jewish population. Conversely, the number of reported patients with these diseases has been limited in Asian countries, including Japan. Here, we reviewed the allele frequencies of pathogenic variants causing ASMD and GD in the Japanese population and populations with various ancestry backgrounds using the Japanese Multi-Omics Reference Panel 54KJPN and the Genome Aggregation Database v4.0.0. The estimated carrier frequencies of ASMD- and GD-related variants were 1/180 and 1/154 in Japanese individuals, equivalent to disease occurrence frequencies of 1/128,191 and 1/94,791 individuals, respectively. These frequencies are much higher than previously expected. Our data also suggest that there are more patients with a milder form of ASMD and nonspecific clinical findings who have not yet been diagnosed.

## High Prevalence of ASMD and GD Variants in Japanese Population

Understanding the frequency of uncommon genetic disorders like Gaucher disease and acid sphingomyelinase deficiency is important, especially in areas where data is limited. In Japan, the actual number of these diseases is still unknown despite medical progress. Researchers focused on identifying harmful variants of the SMPD1 and GBA1 genes, which are associated with acid sphingomyelinase deficiency and Gaucher disease. This review uses existing genetic data from 54,302 Japanese people and compares it with global data to estimate disease frequency in Japan. The main findings show a higher-than-expected frequency of acid sphingomyelinase deficiency and Gaucher disease in Japan, suggesting that many patients may not be diagnosed due to the rarity and nonspecific symptoms of these diseases. Researchers conclude that their work could increase disease awareness and diagnosis, potentially leading to better treatment results for affected individuals.

This summary was initially drafted using artificial intelligence, then revised and fact-checked by the author.

## Introduction

Diagnosing lysosomal storage diseases (LSDs) typically involves enzyme activity measurement, genetic testing, and clinical assessment. However, the diagnosis of acid sphingomyelinase deficiency (ASMD), also called Niemann-Pick disease (NPD) type A, B, or A/B, and Gaucher disease (GD) can be challenging due to their rarity and nonspecific clinical complications, including hepatosplenomegaly and thrombocytopenia^1,2^. Patients with severe subtypes, such as NPD type A and GD type 2, that exhibit neurological involvement have high mortality rates^1,2^. Interestingly, GD types 2 and 3, the severe subtypes, are more common, accounting for more than 50% of GD patients in Japan^3,4^, possibly biasing incidence rates. Moreover, there may be undiagnosed patients with subtle or nonspecific clinical findings. Even with the increasing need for accurate diagnosis of these disorders, carrier frequencies in Japan remain uncharacterized despite advancements in therapeutic approaches, such as olipudase alfa for ASMD and eliglustat for GD^5,6^. We sought to estimate the frequencies of GD and ASMD in Japan using publicly available genome databases.

## Methods

We reviewed the variant allele frequencies (VAFs) of highly pathogenic *SMPD1* and *GBA1* variants in Japanese population and populations with various ancestry backgrounds using two genomic control databases: the Japanese Multi-Omics Reference Panel 54KJPN (jMorp, https://jmorp.megabank.tohoku.ac.jp/, containing genomic data from 54,302 Japanese individuals) and the Genome Aggregation Database v4.0.0 (gnomAD, https://gnomad.broadinstitute.org/, containing genomic data from 807,162 individuals). In gnomAD, we used only a subset of the variant data that passed a quality control filter in the exome. For estimating the prevalence, “DM (disease-causing)” variants reported in patients affected with ASMD and GD entered into the Human Gene Mutation Database (HGMD Professional 2023.4, http://www.hgmd.org/) were defined as pathogenic variants. Additionally, we included potential novel loss-of-function variants, such as pathogenic, frameshift, nonsense, initiation codon, and canonical ± 1 or 2 spice site variants, in *SMPD1* and *GBA1*, even those not registered in the HGMD. Variants classified as benign in the ClinVar database (https://www.ncbi.nlm.nih.gov/clinvar/) were excluded. Small insertion and deletion *SMPD1* variants on chr11:6391620_6391652 (GRCh38/hg38) were not included in this study because the quality values of variants in this region were all low in jMorp, indicating that the mapping process could fail. The VAFs of the pathogenic variants were summed for each gene. The carrier and disease frequencies were calculated as 2pq and q^2^, respectively, based on the Hardy‒Weinberg equilibrium (p = allele frequency of wild-type individuals, q = VAF) using the total VAF^7^.

## Results

In the Japanese population, 24 variants in *SMPD1* and 29 variants in *GBA1* from jMorp were identified as pathogenic according to our criteria (Tables 1 and 2). The most common variant type was missense—16 of 24 *SMPD1* variants (66.7%) and 22 of 29 *GBA1* variants (75.9%). Pathogenic *SMPD1* variants that are commonly reported worldwide, including p.Arg610del, p.Arg498Leu, and p.Phe333Serfs*52, were not included in jMorp^8,9^. Patients with mild forms of ASMD can have a natural history of morbidity extending into their 60s^10^. Considering that 88.1 million individuals in the Japanese population were under 65 years old in 2023, the estimated number of ASMD patients is 687, which is much larger than expected. Notably, the 5 variants with the highest VAFs have been reported in patients with NPD type B, regardless of zygosity (Table 1). p.Pro186Leu, p.Gly244Arg, and p.Arg602His were also identified in NPD type B patients, who are known to have a high residual acid sphingomyelinase activity of 13-43%^11^. A few pathogenic variants associated with NPD type A or A/B were identified.

**Table 1:** Allele frequencies of 24 pathogenic variants in *SMPD1* among Japanese population from jMorp.

Nucleotide change	Protein change	VAF (%)	NPD type of the reported subjects	References
c.592G>C	p.Ala198Pro	0.001148 (41.1)	B; Homo B; Comp het with p.Asp51Val, p.Cys159Arg, p.Gly247Ser, p.Arg291His, p.Arg378His, or p.Cys433Arg	Simonaro et al.^20^
c.995C>G	p.Pro332Arg	0.000387 (13.9)	B; unknown	Simonaro et al.^20^
c.940G>A	p.Val314Met	0.000359 (12.9)	B; Homo B; Comp het with p.Ser192Alafs*65	Lipiński et al.^21^, Desnick et al.^22^
c.340G>A	p.Val114Met	0.000267 (9.6)	B; Comp het with p.His556Tyr	Gucev et al.^23^
c.1133G>A	p.Arg378His	0.000193 (6.9)	B; Comp het with p.Gly168Arg, or p.Ala198Pro	Pavlu-Pereira et al.^24^, Simonaro et al.^20^
c.1106A>G	p.Tyr369Cys	0.000120 (4.3)	A; Homo A/B; unknown	Mercati et al.^25^, Hu et al.^26^
c.872G>A	p.Arg291His	0.000046 (1.6)	B; Comp het with p.Gly168Arg, or p.Ala198Pro	Lipiński et al.^21^, Simonaro et al.^20^
c.557C>T	p.Pro186Leu	0.000037 (1.3)	B; Comp het with p.Gln294Lys	Pavlu-Pereira et al.^24^
c.1708C>T	p.Gln570*	0.000037 (1.3)	Not reported	–
c.1805G>A	p.Arg602His	0.000037 (1.3)	B; Comp het with p.Trp277Arg, p.His516Arg, or p.Arg610del	Simonaro et al.^20^, Cerón-Rodríguez et al.^27^, Zhou et al.^28^
c.2T>A	p.Met1?	0.000018 (0.6)	B; Homo	Pittis et al.^29^
c.688C>T	p.Arg230Cys	0.000018 (0.6)	A/B; unknown B; unknown	Simonaro et al.^20^, Hu et al.^26^
c.689G>A	p.Arg230His	0.000018 (0.6)	B; Comp het with p.Ala597Profs*7	Pavlu-Pereira et al.^24^
c.1216C>T	p.Gln406*	0.000018 (0.6)	Not reported	–
c.61C>T	p.Gln21*	0.000009 (0.3)	B; Comp het with p.Arg610del	Simonaro et al.^20^
c.730G>A	p.Gly244Arg	0.000009 (0.3)	B; Comp het with p.Asn385Ser	Takahashi et al.^30^
c.1026G>C	p.Trp342Cys	0.000009 (0.3)	B; Comp het with p.Arg498Cys	Zhang et al.^31^
c.1231G>T	p.Glu411*	0.000009 (0.3)	Not reported	–
c.1264-2A>G	–	0.000009 (0.3)	Not reported	–
c.1273dupA	p.Ile425Asnfs*24	0.000009 (0.3)	Not reported	–
c.1314C>A	p.Ser438Arg	0.000009 (0.3)	B; Homo	Takahashi et al.^16^
c.1343A>G	p.Tyr448Cys	0.000009 (0.3)	A; Homo A/B; unknown B; Comp het with p.Arg610del	Cerón-Rodríguez et al.^27^, Hu et al.^26^
c.1471dupA	p.Ile491Asnfs*4	0.000009 (0.3)	Not reported	–
c.1474G>A	p.Gly492Ser	0.000009 (0.3)	B; Comp het with p.Gly247Ser	Irun et al.^32^
Total VAF		0.002937 (100)		
Carrier frequency		1 in 180		
Disease frequency		1 in 128,191		

*Comp het* compound heterozygote, *Homo* homozygote, *NPD* Niemann-Pick disease, *VAF* variant allele frequency.

*SMPD1*; NM_000543.5.

**Table 2:** Allele frequencies of 29 pathogenic variants in *GBA1* among Japanese population from jMorp.

Nucleotide change	Protein change	VAF (%)	VAF% in GD patients from Eto et al., [1999]^12^
c.1483G>C	p.Ala495Pro^a,b^	0.001179 (36.3)	–
c.1448T>C	p.Leu483Pro^a,b^	0.000801 (24.7)	41
c.754T>A	p.Phe252Ile	0.000295 (9.1)	14
c.475C>T	p.Arg159Trp	0.000175 (5.4)	–
c.1342G>C	p.Asp448His^a^	0.000157 (4.8)	5
c.1447_1466del20insTG^c^	p.Leu483_Met489delinsTrp	0.000101 (3.1)	3
c.680A>G	p.Asn227Ser	0.000083 (2.6)	1
c.1255G>A	p.Asp419Asn	0.000064 (2.0)	–
c.115+1G>A	–	0.000046 (1.4)	1
c.586A>C	p.Lys196Gln	0.000046 (1.4)	–
c.702dupG	p.Ser235Valfs*27	0.000037 (1.1)	–
c.1448T>G	p.Leu483Arg	0.000037 (1.1)	–
c.1354A>C	p.Lys452Gln	0.000028 (0.9)	2
c.1603C>T	p.Arg535Cys	0.000028 (0.9)	5
c.1193G>T	p.Arg398Leu	0.000018 (0.6)	–
c.1246G>A	p.Gly416Ser	0.000018 (0.6)	1
c.1300C>T	p.Arg434Cys	0.000018 (0.6)	–
c.1528dupA	p.Thr510Asnfs*54	0.000018 (0.6)	–
c.681T>G	p.Asn227Lys	0.000009 (0.3)	–
c.721G>A	p.Gly241Arg	0.000009 (0.3)	–
c.762-2A>G	–	0.000009 (0.3)	–
c.887G>A	p.Arg296Gln	0.000009 (0.3)	–
c.1052G>C	p.Trp351Ser	0.000009 (0.3)	–
c.1192C>T	p.Arg398*	0.000009 (0.3)	–
c.1213A>G	p.Ser405Gly	0.000009 (0.3)	3
c.1225-2A>G	–	0.000009 (0.3)	–
c.1495G>A	p.Val499Met	0.000009 (0.3)	–
c.1504C>T	p.Arg502Cys	0.000009 (0.3)	1
c.1601G>A	p.Arg534His	0.000009 (0.3)	–
The other variants	–	–	23
Total VAF		0.003248 (100)	100
Carrier frequency		1 in 154	
Disease frequency		1 in 94,791	

^a^RecNcil; p.Leu483Pro, p.Ala495Pro, and p.Val499= in *cis.*

^b^RecTL; p.Asp448His, p.Leu483Pro, p.Ala495Pro, and p.Val499= in *cis.*

^c^This variant was separately shown as “c.1447_1462del” and “c.1465_1466del” in jMorp.

*GBA1*; NM_001005741.3.

The spectrum of *GBA1* variants included in jMorp was similar to that identified in Japanese GD patients reported by Eto et al. (Table 2)^12^. The VAFs of the common variants p.Leu483Pro, p.Phe252Ile, and p.Asp448His, which have been observed in homozygous GD type 3 patients, were high in jMorp, reflecting the high prevalence of GD type 3 in Japan^13,14^. Our data predict that there are 929 GD patients among the Japanese population under 65 years of age, which is 7.5 times greater than the 124 patients clinically diagnosed in Japan as of 2009^15^. However, this estimation has limitations because it does not account for the mortality rates of all GD types.

## Discussion

ASMD and GD have diverse incidence rates worldwide, and our data confirmed this diversity (Supplementary Table S1). ASMD is more prevalent in Ashkenazi Jewish and Middle Eastern populations, and the incidence rate of GD is also much greater (1 in 1,124, as estimated previously) in Ashkenazi Jewish people due to a founder effect of the p.Asn409Ser variant (Table 3 and Supplementary Tables S2 and S3)^7^. We found that the spectrum of the *SMPD1* and *GBA1* variants in Japan was distinct from that in populations with high frequencies except for East Asians, suggesting that the Japanese population has a unique variant distribution in ASMD and GD. The variant spectra in the East Asian population were similar to those in Japan. Specifically, 41.7% (10 out of 24 variants) of the pathogenic variants in *SMPD1* and 34.5% (10 out of 29) of those in *GBA1* found in Japanese people were also present in East Asian population in the gnomAD database, which may include some contributions from individuals of Japanese descent (Supplementary Tables S2 and S3).

**Table 3:** Estimated frequencies of ASMD and GD in Ashkenazi Jewish and Middle Eastern populations from gnomAD.

Population	Ashkenazi Jewish	Middle Eastern
*SMPD1*		
Total VAF	0.00859	0.00897
Carrier frequency	1 in 59	1 in 56
Disease frequency	1 in 13,546	1 in 12,432
*GBA1*		
Total VAF	0.03215	0.00309
Carrier frequency	1 in 16	1 in 162
Disease frequency	1 in 967	1 in 104,587

*VAF* variant allele frequency.

A nationwide survey revealed that only three ASMD patients were living in Japan as of 2021^4^. Our study revealed a gap between the number of identified patients and the calculated disease frequencies, which could be explained by patients who have yet to be identified. One important observation was the high frequencies of the variants associated with the milder form of nonneuronopathic NPD type B. Compared to hepatosplenomegaly, other accompanying symptoms, such as lung infiltration and thrombocytopenia, may develop later or are not significant enough to be recognized until hepatosplenomegaly is detected^8^. It is speculated that unidentified patients may have hepatosplenomegaly with other symptoms that are not severe enough to receive medical attention or to consider ASMD as a part of a differential diagnosis. Variants such as p.Ser438Arg, p.Ser233Pro, and p.Ala601Thr have been previously identified in Japanese patients with NPD type B^16,17^. However, these variants were extremely rare or not listed in jMorp, suggesting that they are sporadic private variants.

Many patients with GD were found to have known recombinant alleles harboring the p.Asp448His, p.Leu483Pro, and p.Ala495Pro variants in *cis* by homologous recombination between the *GBA1* gene and the *GBAP1* pseudogene^18^. This event could have resulted in a falsely higher estimated frequency of GD in this study. However, a trial of newborn screening for GD in a local prefecture in Japan revealed that GD occurred in 1 out of 77,720 births, which is close to our result of 1 in 94,791^19^.

This study has limitations, including functionally unverified novel presumable loss-of-function variants and variants annotated as “uncertain significance” or not registered in ClinVar. Because of this limitation, some benign variants unrelated to the disease may have been included in our calculations, even if they were identified in ASMD or GD patients. Additionally, copy number variations and novel missense variants of unknown significance were excluded from the calculations. Despite these constraints, the infrequent occurrence of these variants suggests that our results remain largely unaffected. The prevalence of autosomal recessive inheritance disorders can be influenced by genetic factors, including carrier frequency, founder effects, and consanguinity. However, our findings suggested that the perceived prevalence based on the clinical diagnosis rate may be influenced by other factors, such as lack of awareness of the diseases. The use of genome databases is crucial in modern medicine for determining true disease prevalence. Our findings could increase disease awareness, aiding in identifying more patients who may benefit from emerging therapies. Moreover, this knowledge may prompt physicians to consider ASMD and GD in patients with nonspecific clinical symptoms, such as hepatosplenomegaly and thrombocytopenia.

## Supplementary information

Supplementary Table 1

Supplementary Tables 2, 3

## Data Availability

The data are available from the corresponding authors upon reasonable request.
