# Supplementary material for: Allele frequency of pathogenic variants causing acid sphingomyelinase deficiency and Gaucher disease in the general Japanese population
Source: Hum Genome Var. 2024 Jun 12;11:24. doi: 10.1038/s41439-024-00282-z (PMC11169237; doi:10.1038/s41439-024-00282-z)
Supplement: Supplementary file 1 — Supplementary Table 1 [file 41439_2024_282_MOESM1_ESM.doc]

**Supplementary Table S1.** Estimated carrier and disease frequencies in *SMPD1* and *GBA1* in the gnomAD

| Population | African and AA | Admixed American | European (non-Finnish) | European (Finnish) | Amish |
| --- | --- | --- | --- | --- | --- |
| *SMPD1* |  |  |  |  |  |
| Total VAF | 0.00538 | 0.00576 | 0.00848 | 0.00147 | nd |
| Carrier frequency | 1 in 93 | 1 in 87 | 1 in 59 | 1 in 341 | nd |
| Disease frequency | 1 in 34,501 | 1 in 30,128 | 1 in 13,908 | 1 in 463,634 | nd |
|  |  |  |  |  |  |
| *GBA1* |  |  |  |  |  |
| Total VAF | 0.00609 | 0.00459 | 0.00392 | 0.00152 | 0.01762 |
| Carrier frequency | 1 in 83 | 1 in 109 | 1 in 128 | 1 in 330 | 1 in 29 |
| Disease frequency | 1 in 26,988 | 1 in 47,382 | 1 in 65,112 | 1 in 435,247 | 1 in 3,221 |

| Population | East Asian | South Asian | Others | Total |
| --- | --- | --- | --- | --- |
| *SMPD1* |  |  |  |  |
| Total VAF | 0.00445 | 0.00436 | 0.00827 | 0.00760 |
| Carrier frequency | 1 in 113 | 1 in 115 | 1 in 61 | 1 in 66 |
| Disease frequency | 1 in 50,543 | 1 in 52,557 | 1 in 14,610 | 1 in 17,303 |
|  |  |  |  |  |
| *GBA1* |  |  |  |  |
| Total VAF | 0.00298 | 0.00358 | 0.00551 | 0.00454 |
| Carrier frequency | 1 in 168 | 1 in 140 | 1 in 91 | 1 in 110 |
| Disease frequency | 1 in 112,478 | 1 in 78,136 | 1 in 32,916 | 1 in 48,565 |

*AA* African American*, nd* not detected, *VAF* variant allele frequency
